# Knowledge, beliefs, perceptions and barriers related to implementing smoke-free home practices in two rural settlement areas in Malaysia

**DOI:** 10.18332/tpc/195460

**Published:** 2024-11-22

**Authors:** Siti Nurhasyimah Ayuni Kamni, Nur Ain Nadhirah Binti Saiful Bahron, Aziemah Zulkifli, Isabelle Uny, Rachel O’Donnell, Yayi Suryo Prabandari, Bagas Suryo Bintoro, Emilia Zainal Abidin, Sean Semple

**Affiliations:** 1 Department of Environmental and Occupational Health, Faculty of Medicine and Health Sciences, Universiti Putra Malaysia, Serdang, Malaysia; 2 Institute for Environment and Development (LESTARI), Universiti Kebangsaan Malaysia, Bangi, Malaysia; 3 Institute for Social Marketing and Health, Faculty of Health Sciences and Sport, University of Stirling, Stirling, United Kingdom; 4 Department of Health Behaviour, Environment, and Social Medicine, Faculty of Medicine, Public Health and Nursing, Universitas Gadjah Mada, Yogyakarta, Indonesia; 5 Center of Health Behaviour and Promotion, Faculty of Medicine, Public Health and Nursing, Universitas Gadjah Mada, Yogyakarta, Indonesia

**Keywords:** secondhand smoke, smoke-free home, rural population, tobacco products

## Abstract

**INTRODUCTION:**

Studies have found that parental smoking is the primary source of secondhand smoke (SHS) exposure among children, leading to respiratory illnesses, especially in non-smokers like children and women. Promoting a smoke-free home (SFH) is essential, especially among rural populations, and barriers or challenges to creating a SFH need to be better understood. This study aimed to determine the knowledge levels on SHS and to identify the beliefs, perceptions, barriers and facilitators of SFH practices among the rural population in Kuala Kubu Bharu, Selangor, Malaysia.

**METHODS:**

This study employed a mixed-methods design, conducted in two rural settlement areas in 2022. Data were collected through surveys on SHS and SFH knowledge and face-to-face interviews using a topic guide. The quantitative data were analyzed using SPSS software while the qualitative data were analyzed using the thematic approach via NVivo 12.

**RESULTS:**

Sixty participants completed the survey. Most of the respondents had a good (38%) or moderate (48%) knowledge level of SHS. No association was found between sociodemographic factors and knowledge level. Seven of the nine interviewees knew specific SHS-related health risks. Most participants believed that implementing SFH requires quitting or reducing smoking. Barriers to establishing a SFH included personal convenience, habits, attitudes, and social influence. Family encouragement, practicability, government, and quitting smoking were the facilitators for SFH.

**CONCLUSIONS:**

These rural communities had moderate knowledge level of SHS and SFH. Men’s knowledge, beliefs and perceptions like associating SFH with quitting smoking may prevent SFH adoption. It is critical for the government and stakeholders to disseminate information and develop socially and culturally acceptable health promotion programs, incorporating the considerations from this study to increase the chances of SFH implementation in rural areas.

## INTRODUCTION

There is no safe threshold for secondhand smoke (SHS) exposure; even minimal exposure may lead to acute damage^1^. SHS is one of the major sources leading to poor indoor environment quality and is estimated to cause nearly 0.9 million attributable deaths per year and approximately 0.7% of all global morbidity^2^. Several studies have found that parental smoking is the primary source of children’s SHS exposure. Sudden infant death syndrome, acute respiratory infections, ear troubles and more severe asthma are all elevated hazards for children exposed to SHS especially in the home^3^. There is a wide range of prevalence rates for children living with smoking parents^4^ and approximately 44% of children reported living with one or more smokers in the UK^5^. Regional data show SHS exposure in Sri Lanka as 17.6%^6^ and almost two-thirds of infants live with fathers who are smokers in Indonesia^7^. Data from Malaysia indicate that more than one-third of children are exposed to SHS at home^8^. Therefore, creating a smoke-free environment to protect non-smokers, especially children from exposure to SHS, may be seen as the best option^9^.

Overall prevalence of smoking is 21.3% or approximately 4.8 million smokers in Malaysia, making it one of the highest in the Asian region^10^. Smoking prevalence is higher among males (40.5%) than females (1.2%), and higher among rural residents (25.4%) compared to urban residents (20.1%)^11^. Malaysia has ratified the WHO Framework Convention on Tobacco Control (FCTC) in 2005, which results in the prohibition of smoking in public spaces and on public transport. In line with Article 8 of the FCTC, multiple initiatives have been undertaken by the health officials to protect the Malaysian public from SHS exposure, among others the MQuit and *Jom* Quit smoking cessation program and Tak Nak! anti-tobacco program^12^. MyHouse program, which was introduced in 2021, was a latest initiative to the Generational End Game to reduce SHS exposure among the public. These programs aim to promote smoking cessation services; however, their effectiveness may not be easily attainable, as seen from the persisting smoking prevalence trend^10^ and data showing the lack of smoking restriction practices in the home^13^. In other countries, observational studies link SFH rules to higher quit rates^14,15^ and reduced smoking uptake in adolescents^1,16^. As such, the concept of encouraging SFH may be a highly valuable approach which can be leveraged more significantly.

Qualitative studies on behavior change related to SFH shows evidence of barriers faced by households in establishing SFH; these revolved around the seasoned habit of smoking indoors and lack of knowledge with regard to ill-health^1,16^. Health, specially protecting the health of children is the most important motivator for implementing SFH, and multiple reviews have focused on enablers which are defined as person, agency, strategy or other assistance that might help households to create or maintain an SFH^17^. However, these studies were conducted in countries where there is a decline in the smoking trend and comprehensive smoke-free legislation has been implemented^18,19^, unlike in Malaysia.

Health promotion initiatives must consider that social and cultural norms affect SFH barriers, motivators and enablers^20^. Our previous work found that male smokers in urban areas linked SFH hurdles to their inability to quit smoking^21^. There were relatively higher concerns about the smell of SHS instead of its impacts on health. Specific efforts are needed to develop scalable strategies for encouraging smoking prohibition in the home to enhance SFH implementation especially in rural areas where smoking prevalence is greater^14^. Protecting populations at risk from continued SHS exposure in the home is an important aim and is aligned with the Malaysian National Strategic Plan for Tobacco Control 2021–2025^22^. This study fills a gap in the literature where further studies focusing on Malaysians’ views, perceptions and challenges to smoke-free household implementation especially in the rural setting are needed. The aims of this study were to determine the knowledge level on SHS and to identify beliefs, perceptions and barriers for establishing an SFH among a rural population in Malaysia.

## METHODS

This study was conducted among rural populations in two agricultural settlement areas in Selangor, West Malaysia. The settlement areas are under the Federation of Land Development Agency (FELDA), a government agency focusing on relocating rural poor populations to newly developed settlements to perform agricultural activities such as cash crops^23^. These study areas are adjacent to each other with a combined population of approximately 3400. Typically, homes are built using brick and concrete on plots approximately 1000 m^2^, offering ample space for planting^24^. Data collection was carried out between December 2022 and January 2023, after the study received ethical approval from Institutional Review Board with ID number JKEUPM-2022-920 and subsequent approvals from the FELDA officials.

### Study design

This study combined both quantitative and qualitative methods, involving a survey of the population and in-depth interviews with a sample of respondents.

### Quantitative methodology

*Questionnaire*

A questionnaire with closed-ended questions on knowledge level of SHS and SFH, taking around 15 minutes to complete, was used for quantitative data collection. The sociodemographic section of the questionnaire covered the following characteristics: gender, age, race, religion, education level, average monthly household salary, household background and smoking status. The second section contained a 15-item list of statements used to assess knowledge of SHS and SFH with response options assessed as ‘correct’, ‘incorrect’ or ‘not sure’. Nine of the statements were adapted from a previously validated questionnaire^25^, the remaining six statements were developed from the literature^13,21^. To ensure the validity of the questionnaire, two experienced Public Health researchers were asked to evaluate the items in the questionnaires on their relevance to SHS and SFH, and to determine their clarity. The evaluation results were then used to improve the questionnaire. The reliability of the final questionnaire was measured using Cronbach’s alpha, resulting in a value of 0.65, which indicates a satisfactory level of internal consistency.

*Recruitment and sampling*

Data were collected using face-to-face methods where researchers recruited community members at key public gathering spots where individuals frequently convened. Other approaches were employed to broaden the number of participants, including recruitment through the head of the village, displaying posters for advertisements and utilizing ‘snowball’ recruitment by reaching out to previous contacts. Interested participants were given information sheets and the opportunity to ask questions, prior to providing informed consent to take part. The information and communication were delivered in Malay. Respondents were invited to participate if they met the inclusion criteria, which included being aged ≥18 years and living in the surrounding area. From an initial pool of 84 potential respondents, 24 were found to be ineligible based on the inclusion criteria. A total of 60 participants were recruited for the quantitative section of the study.

*Analysis*

The collected questionnaires were screened, and data were entered into Statistical Packages for Social Sciences (SPSS) version 27.0 for further analysis. Correct responses to each statement were given 1 point, while incorrect or ‘don’t know’ responses were given a 0 point. By combining the scores for each respondent into a total score, a knowledge score was calculated, which had a range from 0 to 16, and was then changed to a percentage. Cut-off points were determined to categorize knowledge as follows^26^ : poor knowledge corresponds to <50% of the total score, satisfactory knowledge is 50–75% of the total score, and good knowledge is >75% of total score. Based on these criteria, the knowledge scores calculated in this survey were classified as good knowledge (score >12), satisfactory knowledge (score 8–12) or poor knowledge (score <8). Fisher’s exact test was then used to determine the association between the sociodemographic variables and the knowledge level of SHS and SFH among the population.

### Qualitative methodology

Qualitative interview schedules were adapted from previous work conducted by members of the research team^21^.

### Recruitment and sampling

The participants were first screened before being recruited. The inclusion criteria included male smokers (of combustible cigarettes) aged ≥18 years and living with at least one child (or grandchildren) aged ≤16 years at home and had a habit of smoking inside or immediately outside of the home (for example on the veranda). Those who used electronic cigarettes were considered ineligible to participate in the study. A total of 9 participants were recruited for this section of the study following recommendations from the literature^27^.

Interview questions explored topics including: 1) smoking beliefs and perceptions, 2) barriers, and 3) enablers to creating and maintaining an SFH. The interviews lasted on average 20–25 minutes and audio recorded for analysis purposes. The researcher encouraged respondents not to include family members during the interview process to minimize potential bias in their answers.

*Analysis*

The audio recordings were transcribed into text and translated into English from Malay by SNAK and NANSB. Anonymized transcriptions were uploaded into NVivo 12 for coding. SNAK, NANSB and EZA conducted thematic analysis, which entails searching across a set of data to identify existing patterns^28^. Thematic analysis was employed, utilizing both: 1) a deductive approach by reviewing research questions and topic guides, and 2) an inductive approach derived from repeated reading and analysis of the transcripts. The framework approach and framework matrices from Nvivo were utilized to summarize data under each theme. The summaries were then synthesized accordingly.

## RESULTS

### Questionnaire survey

In total, 60 respondents were recruited and aged 19–66 years. The majority of the respondents (52%) were aged >50 years and most were males (78%). Participants were almost exclusively of Malay ethnicity (95%) and most had completed secondary school (58%). Four out of five (80%) respondents had an average household monthly income of <4360 MYR (1000 Malaysian Ringgits about US$220). Smoking was only reported among male respondents, (62%, n=47) and the highest number of cigarettes smoked per day was between 6 to 20 cigarettes. Table 1 presents the sociodemographic distribution of the respondents from the two study areas.

**Table 1:** Sociodemographic characteristics of participants from two settlement areas in Malaysia, 2022 (N=60)

*Characteristics*	*Total (N=60) n (%)*	*Felda I (N=30) n (%)*	*Felda II (N=30) n (%)*
**Age** (years)			
18–29	11 (18.3)	1 (3.3)	10 (33.3)
30–49	18 (30)	12 (40)	6 (20)
>50	31 (51.7)	17 (56.7)	14 (46.7)
**Gender**			
Male	47 (78.3)	23 (76.7)	24 (80)
Female	13 (21.7)	7 (23.3)	6 (20)
**Ethnicity**			
Malay	57 (95.0)	30 (100)	27 (93.3)
Other	3 (5.0)	0 (0)	3 (6.7)
**Religion**			
Islam	57 (95.0)	30 (100)	27 (93.3)
Other	3 (5.0)	0 (0)	3 (6.7)
**Education level**			
Primary	13 (21.7)	7 (23.3)	6 (20)
Secondary	35 (58.3)	14 (46.7)	2 (70)
Tertiary	12 (20.0)	9 (30)	3 (10)
**Monthly income** (MYR)			
≤1500	26 (43.3)	13 (43.3)	13 (43.3)
1501–4360	22 (36.7)	10 (33.3)	12 (40)
4361–9619	6 (10.0)	2 (6.7)	4 (13.4)
≥9620	6 (10.0)	5 (3.3)	1 (3.3)
**Children in household**			
Yes	24 (40.0)	14 (46.7)	10 (33.3)
No	36 (60.0)	16 (53.3)	20 (66.7)
**Pregnant women in household**			
Yes	3 (5.0)	2 (6.67)	1 (3.33)
No	57 (95.0)	28 (93.3)	29 (96.7)
**Active smoker**			
Yes	29 (48.3)	15 (50)	14 (46.7)
No	31 (51.7)	15 (50)	16 (53.3)
**Cigarettes per day**			
1–5	9 (34.6)	5 (33.3)	4 (36.3)
6–20	11 (42.3)	7 (46.7)	4 (36.3)
21–30	6 (23.1)	3 (20)	3 (27.3)
**Smoking area**			
Living room/bedroom	11 (37.9)	5 (33.3)	6 (42.9)
Toilet inside	2 (6.9)	1 (6.7)	1 (7.1)
Veranda	6 (20.7)	1 (6.7)	5 (35.7)
Outside of home	10 (34.5)	8 (53.3)	2 (14.3)

MYR: 1000 Malaysian Ringgits about US$220.

In terms of knowledge of SHS, the mean score was 11.40 (standard deviation, SD=2.90). When the scores were categorized into groups of good, satisfactory and poor, the results showed that the respondents generally had a good (43.3%, n=26) or moderate (satisfactory) (43.3%, n=26) level of knowledge on SHS. Only 8 participants (13.3%) were assessed as having poor knowledge. The distribution of knowledge scores regarding SHS among the population of the rural areas is shown in Table 2.

**Table 2:** Knowledge of participants about secondhand smoke and smoke-free homes among participants in two settlement areas in Malaysia, 2022 (N=60)

*Variables*	*Correct n (%)*	*Incorrect/don't know n (%)*
SHS is smoke that has been exhaled, or breathed out, by the person smoking from burning tobacco products, like cigarettes, cigars, hookahs, or pipes.	15 (30.0)	45 (70.0)
Inhaling smoke from someone else’s cigarette is harmful to one’s health.	56 (93.3)	4 (6.7)
SHS contains substances known to be toxic.	51 (86.7)	9 (13.3)
There is no safe level of exposure to SHS.	44 (73.3)	16 (26.7)
SHS has the same harmful chemicals that people who smoke inhale.	35 (50.0)	25 (50.0)
There have been a number of cases in which non-smokers developed lung cancer because of exposure to SHS.	47 (83.3)	13 (16.7)
Exposure to SHS has immediate adverse effect on cardiovascular system.	42 (63.3)	18 (36.7)
Exposure to cigarette smoke is associated with sudden infant death syndrome (SIDS).	33 (53.3)	27 (46.7)
Maternal exposure to SHS during pregnancy causes low birthweight.	37 (56.7)	23 (43.3)
Breathing smoke from other people’s cigarettes cause heart disease in adults.	38 (66.7)	17 (33.3)
Children who are exposed to SHS are at increased risk of having lung illnesses.	41 (60.0)	19 (40.0)
The effect of SHS is greater to the people inside the home than the outside.	54 (90.0)	6 (10.0)
The particles from SHS can settle in dust inside the house and on surfaces and remain there long after smoke is gone	43 (66.7)	17 (33.3)
There is a smoke-free law covering public spaces in Malaysia	59 (96.7)	1 (3.3)
There is a smoke-free home policy in Malaysia	53 (83.3)	7 (16.7)

Correct answers for ‘agree’ responses to the statements. Incorrect answers ‘not agree’ and ‘not sure’ responses to the statements. SHS: secondhand smoke.

The category of knowledge was cross-tabulated across sociodemographic variables of interest. There was no association between the sociodemographic characteristics (age, gender, education level, and monthly income) and the level of knowledge regarding SHS and SFH, except for smoking status where higher knowledge level was associated with active smoking status. Table 3 presents the cross-tabulation between the knowledge level of SHS with sociodemographic characteristics among the population of the two settlement areas (n=60).

**Table 3:** Cross-tabulation of knowledge levels on secondhand smoke and smoke-free homes with sociodemographic characteristics among the participants in two settlement areas in Malaysia, 2022 (N=60)

*Variables*	*Level of knowledge, n (%)*			*p*
	*Poor*	*Fair*	*Good*	
**Age** (years)				0.964
18–29	1 (9.1)	6 (54.5)	4 (36.4)	
30–49	2 (11.1)	8 (44.4)	8 (44.4)	
≥50	5 (16.1)	15 (48.4)	11 (35.5)	
**Gender**				0.425
Male	6 (12.8)	21 (44.7)	20 (42.6)	
Female	2 (15.4)	8 (61.5)	3 (23.1)	
**Education level**				0.684
Tertiary	0 (0)	3 (30.0)	7 (70.0)	
Secondary	5 (13.9)	19 (52.8)	12 (33.3)	
Primary	3 (21.4)	7 (50.0)	4 (28.6)	
**Average monthly income per household (MYR)**				0.395
≤1500	1 (3.8)	14 (53.8)	11 (42.3)	
1501–4360	4 (18.2)	10 (45.5)	8 (36.4)	
4361–9619	3 (25)	5 (41.7)	4 (33.3)	
**Smoking habit**				0.039*
Yes	3 (10.3)	10 (34.5)	16 (55.2)	
No	5 (16.1)	19 (61.3)	7 (22.6)	
**Cigarettes per day**				0.254
1–5	4 (44.4)	4 (44.4)	1 (11.1)	
6–20	1 (9.1)	9 (81.8)	1 (9.1)	
21–30	1 (16.7)	3 (50.0)	2 (33.3)	

Statistic test performed was Fisher’s exact test.

* p<0.05 indicates statistical significance. MYR: 1000 Malaysian Ringgits about US$220.

### Interviews

Nine participants from the two rural populations were interviewed. Table 4 presents the sociodemographic distribution of the respondents in the two settlement areas.

**Table 4:** Sociodemographic distribution of qualitative respondents in two settlement areas in Malaysia, 2022 (N=9)

*Participant ID*	*1*	*2*	*3*	*4*	*5*	*6*	*7*	*8*	*9*
**Age** (years)	38	42	61	67	54	59	48	47	55
**Role in household**	Father	Father	Father	Father	Father	Father	Father	Father	Father
**Children in the household**	2 boys (3 and 4 years) 1 girl (9 years)	1 girl (7 years)	1 boy (14 years)	1 boy (9 years)	1 girl (16 years)	2 boys (3 and 5 years)	1 girl (16 years)	1 boy (7 years)	1 girl (16 years) 1 boy (13 years)
Education level*	Secondary	Secondary	Primary	Primary	Secondary	Secondary	Secondary	Tertiary	Secondary
Household income status**	B40	B40	B40	B40	B40	M40	B40	B40	B40
**Home smoking rules**	Only smoke in office-like room	Only smoke on veranda	No rules	No rules	Smoking limited to some parts of the home	Living room	Living room	Sometimes smoke in the home	Sometimes smoke in living room

* Primary education level: 7–12 years of formal education. Secondary education level: 13–17 years of formal education. Tertiary education level: university.

** B40 is the lower 40% of Malaysia population with monthly household income (MYR) of <4850, M40 is the middle 40% with household income 4850–10970, T20 is the upper 20% with household income of >10970.

*Smokers’ beliefs and perception of SHS exposure and SFH*

Regarding the degree to which SHS exposure in the home poses a health risk, interviewees expressed a variety of opinions. Almost all of them were aware that SHS exposure is dangerous to people nearby:

*‘I’m worried because we, as heavy smokers, are happy to face the risk of anxiety leading to discomfort and pain and so on, worry that people close to you like your wife will get the same risk as I said earlier.’* (Participant 6, usually smoke in the living room)

However, only a few were aware of the specific health risks posed by SHS. Only one expressed clearly that SHS may lead to lung cancer, breathing problems and premature births for pregnant women; other respondents agreed that SHS may cause lung and heart disease:

*‘It’s the lungs. Often these cigarettes will affect the lungs.’* (Participant 5, limiting smoking to some parts of the home)

By contrast two respondents mentioned that they were unaware of any health risks arising from SHS exposure. Several of the respondents expressed concerns about SHS relating it not only primarily to the smell of smoke in the home but to the fact that it can irritate others by affecting their health:

*‘If I smoke on the verandah, it will impact my family’s health because the wind can bring the smoke into the kitchen. I am worried, but this is my habit.’* (Participant 9, only smoke at home sometimes)

*Family members’ perception of SHS exposure in the house*

Three of the respondents said that their wives did not like the smell of cigarette smoke and scolded them for smoking in the house:

*‘My wife really doesn’t like the smell of cigarette smoke. She said, “Give me some space!” [Ordering her husband to smoke far away from her].’* (Participant 1, only smoke in an office-like room inside the house)

However, only one respondent reported that his wife accepted him smoking:

*‘Even my wife doesn’t forbid it. “Smoke as long as you want to smoke …”, she said.’* (Participant 3, no smoking rules in the house)

On the other hand, all the respondents’ children disapproved of their fathers’ smoking behavior. According to one respondent, his daughter appeared to be more assertive in her advice-giving and reprimanding, whereas sons were more prone to do it lightly:

*‘Yes, they got angry, usually my daughter. “If you want to smoke, go outside. I feel like I am drowning”, she said.’* (Participant 5, limiting smoking to some parts of the home).

Despite the advice given by their children, one respondent said it was ignored:

*‘There have been several times (of children complaining) but we are egoistic.’* (Participant 8, smoke in the living room)

*Barriers regarding the implementation of SFH*

The majority of participants explained that there were important barriers to establishing SFH: smoking and habitual behavior, difficulties in quitting, convenience, and social activities.

Habitual behavior and attitude

One respondent said it was his habit to smoke before going to sleep in the bedroom:

*‘Usually, if I want to sleep, I will smoke half of a cigarette, I will daydream first, until I fall asleep. Once I’m sleepy, I throw away the cigarette and go to sleep.’* (Participant 4, no smoking rules in the house)

Another respondent said that attitude could be the reason to accept or reject the idea of practicing SFH:

*‘If you yourself give this kind of explanation to a smoker and the smoker can accept it, it’s easy. But, if the smoker can’t accept it, what should we do?’* (Participant 2, smoke on the veranda)

While the other respondents had the habit of smoking more likely after eating, especially after lunch, and when they were bored.

Inability to quit smoking

Most of the respondents associated SFH with the need to quit smoking completely and identified this as a key barrier. Quitting, for some, was seen as especially difficult since they had been smoking for a long period of time. Several respondents alluded to addiction making it difficult to quit smoking. Meanwhile, other respondents felt that their inability to quit thwarts the possibility of implementing SFH:

*‘I’ve been attempting to quit smoking for two years. But when we see friends smoking, we smoke again, first one stick, then another. People said that when you are addicted, you can’t get rid of it.’* (Participant 4, no smoking rules in the house)

Convenience and comfort

Being able to smoke in comfortable surroundings (i.e. in a comfortable chair) was noted as one of the main reasons a few of the respondents gave for smoking indoors. One of them said:

*‘I also find it difficult because there is no comfortable place to smoke outside. It’s normal for us humans to want something easy, right? So, whatever is close is easy.’* (Participant 2, smoke on veranda off the living room)

Besides, several of the respondents complained that their houses were located close to an oil palm plantation, where a lot of mosquitoes posed a nuisance and caused them to stay inside the house, even when smoking.

Difficulty in enforcing SFH for visitors

Several respondents mentioned that they would have difficulty stopping visitors and guests from their smoking habits when they come to visit their households:

*‘If we want to have a smoke-free house, we can’t have smoking guests come to the house, it’s difficult, do we want to be angry at them for smoking?’* (Participant 1, smoking in an office-like room inside the house)

*Enablers of SFH adoption*

Respondents mentioned a number of factors that could encourage them (or people they knew) to establish SFH. These could be broadly classified as covering factors relating to personal responsibility, their families, the availability of adequate facilities and the role of government.

Personal responsibility and family influence

Several respondents mentioned the need for smokers themselves to take the responsibility to implement SFH. They also acknowledged their role as the head of the family with responsibility for their family’s health and saw it as a possible motivation for fathers to establish SFH:

*‘I’m the only one [who is responsible to create SFH], no one else. If I say don’t smoke, no one can smoke.’* (Participant 5, limiting smoking to some parts of the home)

*‘Actually, for the main role, the head of the family, whether a smoker or a non-smoker, will be able to immediately ban any cigarettes that come in.’* (Participant 6, usually smoke in the living room)

Most of the participants’ family members were also mentioned as having a positive influence in setting up an SFH. Therefore, the perception of the family members could influence the fathers to smoke outside. These scenarios happened in several of the respondents’ families, for example:

*‘Yes, sometimes my wife tells me to smoke outside. When there are small children back at home, I will sit outside. If it’s just me, I’ll just smoke in the living room.’* (Participant 5, limiting smoking to some parts of the home)

A large number of respondents said that they did not smoke when there were small children nearby, and some mentioned that it was due to feelings of guilt. They did walk away to smoke outside, as they knew that, generally, SHS could have a bad health effect on those who were exposed:

*‘I feel uncomfortable smoking in front of my children and wife. Usually I will go far away.’* (Participant 1, only smoke in an office-like room inside the house)

Government’s role

When asked who is responsible for creating a SFH, besides the head of the family, respondents expressed it was the government:

*‘People smoke because cigarettes are sold. If the government doesn’t sell cigarettes, we don’t smoke either. Maybe if there is a smoking ban law, maybe we can force smokers to stop smoking.’* (Participant 2, smoke on veranda off the living room)

*‘It’s hard to get rid of these cigarettes. It also depends on government initiatives. If you forcefully close the cigarette factory, cigarettes are no longer sold in the shop, maybe people will stop smoking cigarettes.’* (Participant ID 03, having no rule of smoking in the house).

Practicability for outdoor smoking

Most of the respondents expressed their thoughts regarding the impracticality of smoking outside the house for several reasons. While some of the reasons for smoking indoors were seen to be due to convenience, as stated above, one of the ways to overcome them was stated as to have a practicable way, spot, or facility to smoke outside the house. This approach not only aligns with the concept of practicability but also offers a viable alternative for smokers and promoting healthier indoor environments for non-smoking family members. One father suggested the following:

*‘Having a proper smoking area with a chair outside that is as comfortable as inside the house may aid in practicing SFH.’* (Participant 2, smoke on veranda off the living room).

## DISCUSSION

This study provides insights regarding the knowledge, beliefs and perceptions on SHS and SFH among a rural agricultural community in Selangor, Malaysia, in addition to other pertinent factors about barriers and enablers to the adoption of SFH. The current study resembled a prior investigation where a significant majority of participants were aware of the potential harmful effects of SHS on health, but with limited knowledge regarding the specific health impacts of inhaling cigarette smoke from others^29^. The qualitative part further revealed that despite everyone believing SHS could harm their children, a few of them did not worry about smoking around others, leading to a lack of SFH practice.

This study found there is a belief that smoking is not always related to diseases and death, but rather it is a matter of fate based on their religious beliefs, besides adhering to some medical tips for safer smoking. Most of the respondents had become accustomed to smoking before or after certain daily activities in specific places, such as after lunch in an office-like room, before sleeping in the bedroom, and during leisure time in the living room. Previous studies^30,31^ have identified smoking by visitors as another obstacle to the adoption of SFH, aligning with our research findings. Young parents faced challenges linked to social and cultural aspects where it is difficult to implement SFH when there are elderly guests with smoking habits^31^. On the other hand, most of the participants smoke at some designated specific areas that are convenient to them, such as office-like rooms and verandas, in the presence of their children, believing that the SHS would not be shared with others in the house. Enforcing SFH in rural areas is not straightforward as in urban areas, due to the discomfort and inconvenience associated with outdoor smoking, particularly in rural communities near palm oil plantations where mosquitos are prevalent, causing smokers to prefer smoking indoors. Also, participants see quitting smoking as necessary for SFH, but they believe their self-esteem to be inadequate to do so, as addiction makes it challenging. This confirms findings from earlier research^21^. Additionally, earlier research involving 18 smoking fathers in Scotland aligns with these findings, indicating that obstacles to establishing a SFH include issues such as struggles with nicotine addiction^20^.

On the other hand, this study also identified enabling factors that became motivations to implement SFH, with comparable findings elsewhere^20^. In the previous work, fathers expressed a strong sense of responsibility to protect their children from harm, representing the ‘father-protector’ role. The need to be a ‘good father’ encouraged them to take measures to restrict their children’s exposure to SHS within the home^21^. A comprehensive scoping review by O’Donnell et al.^32^ found that fathers’ attitudes, knowledge, cultural and social norms, gender power dynamics, and evolving perceptions of fatherhood, influence their role in creating and maintaining a smoke-free home. Besides, the participants who had some kind of smoking restrictions reported that their wives or children disliked the smell of SHS^21^, highlighting the important of children’s viewpoint in shaping SFH restrictions in the home. Interestingly, most of the respondents noted that their daughters were more assertive in giving reprimands or criticisms, signaling women’s roles in providing assertive communication to protect the health of the family.

Researchers noted that social norms in the settlement eateries allow smokers to freely smoke, unlike in cities where public smoking is less tolerated. This supports a respondent’s claim that implementing SFH in villages is harder due to widespread public smoking. A study emphasized that normalization of smoke-free environments in public places can influence smokers to implement SFH due to the shifting social attitudes towards smoking^33^. Finally, the practicality of outdoor smoking is a key factor that can encourage smoking outdoors. This aligns with the study by Odes et al.^34^ which suggested that SFH initiatives such as designated smoking zones outside the house can support indoor smoke-free policy.

The findings from the interviews conducted brings together features described in numerous health promotion model such as roles of intention, perceptions and beliefs about harm, family and influences of peers, self-esteem and other enabling factors such as the role of the government. To better summarize our findings, we used the Health Action Model which provides an integrative perspective and is a useful framework at looking at factors that affect behavior such as roles of intention, beliefs, motivational factors, social-network influence, self-concept and enabling factors^35^. This model suits the present study because it acknowledges the complex influences and factors that exist in dealing with how an individual views SHS and what are the considerations in adopting SFH.

**Figure 1:**
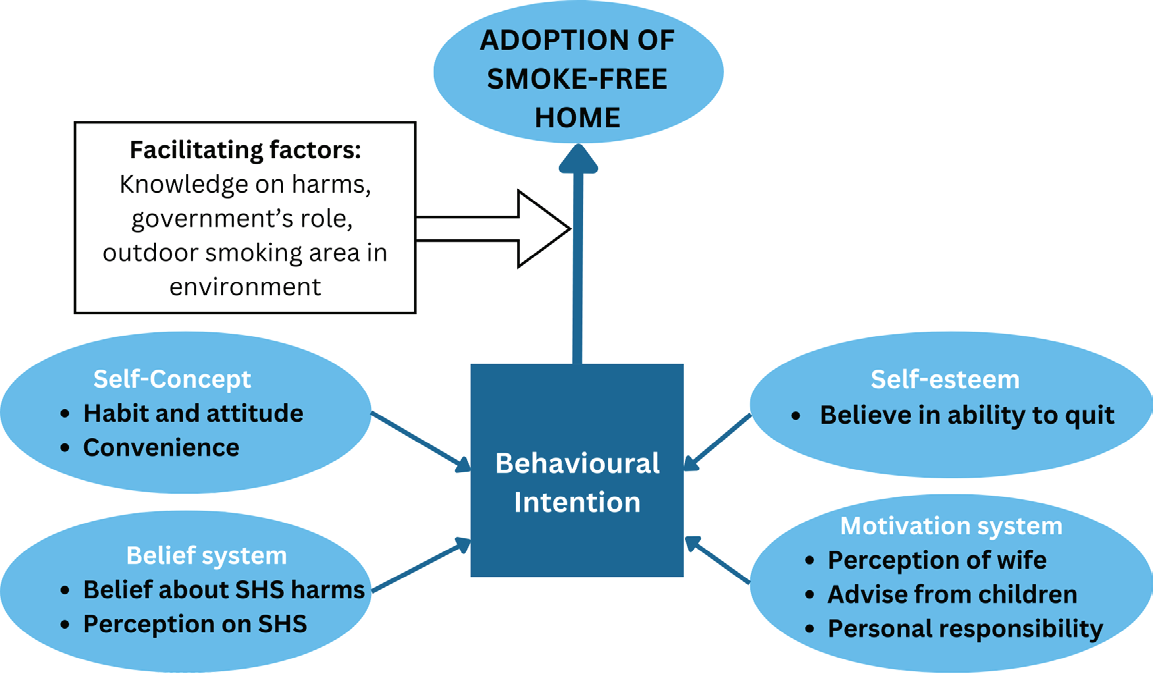
Combination of factors influencing the adoption of SFH among the study population from two settlement areas in Malaysia, based on the Health Action Model, 2022

By adopting the structures of the Health Action Model, a previous study demonstrated significant positive changes in the awareness, attitudes, norms and beliefs of participants following a three-month educational intervention^36^. Our study postulated that to adopt SFH, it requires a combination of factors based on the Health Action Model (Figure 1). This includes the beliefs about the harm caused by SHS and perceptions of its risks. The self-concept which influences a smoker to quit, encompasses habitual behavior and attitudes, as well as convenience and self-esteem, crucial factors in adopting an SFH. The motivational system is also important to the smokers, influenced by factors such as the perception of their wives, advice from the children, and personal responsibility. Finally, facilitating factors like knowledge about the harms of SHS, government support and outdoor smoking areas can lead to the implementation of SFH.

This study highlights the need for SHS and SFH interventions in rural areas, especially among the elderly, incorporating scientific evidence while considering cultural aspects and religious beliefs. The primary challenge in implementing SFH in this rural population seems to stem from the lack of convenient outdoor smoking areas. The village leaders may consider addressing the almost non-existent enforcement of smoke-free law in rural communities, particularly in eateries, with the hope that it will promote the practice of SFH.

### Strengths and limitations

The strength of this study lies in the fact that the results represent data and opinions of a population generally known to be at social disadvantage. By acknowledging the unique challenges, the study can develop a foundation for developing strategic and localized health promotion campaigns. However, the overall sample size for the quantitative part in this study was minimal for an approximate population of 3400 people. Hence, there is a need to be cautious in extrapolating the results to the general population. Instead, it would better represent a unique type of predominantly agriculture community which are present in Malaysia. This study is also among the first to conduct a thematic synthesis on SFH implementation in settlement areas, leaving no local studies to support its conclusions. Future research could build on this by conducting more local investigations with larger sample size to validate and strengthen the findings.

## CONCLUSIONS

This study found that the rural community in two settlement areas had a moderate level of knowledge on SHS and SFH. Men’s knowledge, beliefs and perceptions such as anchoring the idea of SFH with quitting smoking may become a roadblock preventing households from even considering implementing SFH practices. As a result, it is critical for the government and interested parties to develop and disseminate information and health promotion programs in a way that is socially and culturally acceptable, incorporating the considerations found from this study, to increase the chances of SFH implementation in rural areas.

## Data Availability

The data supporting this research are available from the authors on reasonable request.
